# Towards precision radiation oncology: endocrine therapy response as a biomarker for personalization of breast radiotherapy

**DOI:** 10.1038/s41698-023-00348-1

**Published:** 2023-01-24

**Authors:** S. M. Nashir Udden, GuemHee Baek, Kamal Pandey, Chantal Vidal, Yulun Liu, Asal S. Rahimi, D. Nathan Kim, Chika R. Nwachukwu, Ram S. Mani, Prasanna G. Alluri

**Affiliations:** 1 Department of Radiation Oncology, UT Southwestern Medical Center, Dallas, TX 75390 USA; 2 Department of Pathology, UT Southwestern Medical Center, Dallas, TX 75390 USA; 3 Harold C. Simmons Comprehensive Cancer Center, UT Southwestern Medical Center, Dallas, TX 75390 USA; 4 Department of Population and Data Sciences, UT Southwestern Medical Center, Dallas, TX 75390 USA

**Keywords:** Molecular medicine, Breast cancer, Breast cancer

## Abstract

Targeted therapies, such as endocrine therapies (ET), can exert selective pressure on cancer cells and promote adaptations that confer treatment resistance. In this study, we show that ET resistance in breast cancer drives radiation resistance through reprogramming of DNA repair pathways. We also show that pharmacological bromodomain and extraterminal domain inhibition reverses pathological DNA repair reprogramming in ET-resistant breast tumors and overcomes resistance to radiation therapy.

Endocrine therapy (ET) use in the pre-operative setting in localized estrogen receptor (ER)-positive breast cancer (BC) is associated with comparable rates of response and breast preservation relative to chemotherapy but lower toxicity in post-menopausal women^1^. Overall, 20–35% of tumors treated with pre-operative ET show evidence of intrinsic or acquired ET resistance^2,3^. Mutations in the ligand binding domain of gene encoding ER (*ESR1*) represent one of the most common mechanisms for acquired ET resistance in patients with metastatic, ER-positive BC^4^. *ESR1* mutations are also enriched in localized BC patients following pre-operative ET treatment^5^.

To define the impact of acquired ET resistance on response to radiation therapy (RT), we carried out clonogenic survival assays following ionizing radiation (IR) in ER-positive MCF-7 cells and T-47D cells that have been genomically edited to knockin *ESR1* Y537S and D538G mutations. *ESR1* Y537S and D538G are the two most commonly enriched mutations in patients with both metastatic and localized BC following treatment with ET^4,5^. Both MCF-7 and T-47D cells harboring Y537S and D538G mutations exhibited enhanced survival following treatment with IR, suggesting that they are radiation resistant (Fig. 1a–c and Supplementary Fig. 1a, b). Similarly, MCF-7 tamoxifen-resistant (Tam-R) BC cells exhibited higher survival following IR treatment relative to the corresponding parental cells (Supplementary Fig. 1c, d), suggesting that acquired ET resistance in BC also drives radiation resistance (RR). To study if RR in these ET-resistant BC cells is associated with reprogramming of DNA repair pathways, we assessed γ-H2AX foci formation at 30 minutes and 120 minutes following IR treatment. Both MCF-7 and T-47D cells harboring *ESR1* Y537S and D538G mutations exhibited diminished γ-H2AX foci formation at these time points (Fig. 1d, e and Supplementary Fig. 1e, f). These cells also exhibited diminished comet tail moment relative to the corresponding cells harboring the wild-type (WT) *ESR1* (Fig. 1f, g, and Supplementary Fig. 1g, h). These findings suggest that RR in ET-resistant BC cells is associated with a lower degree of unrepaired DNA damage following exposure to IR.

**Fig. 1:**
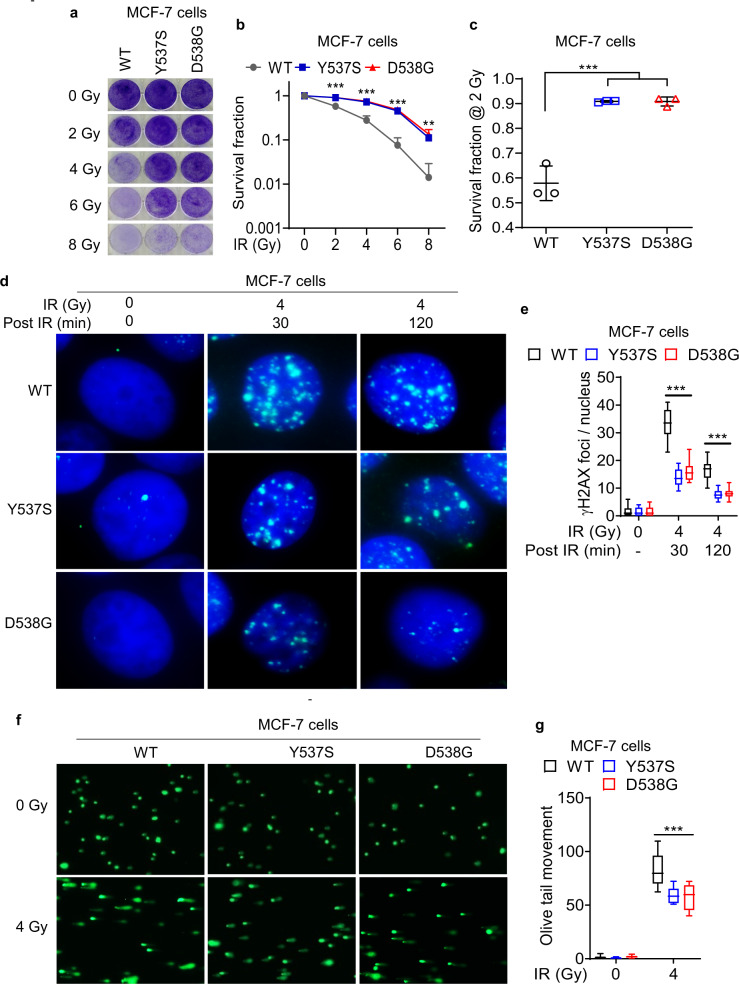
Acquired ET resistance in ER-positive BC cells confers radiation resistance. **a**–**c** ET-resistant MCF-7 Y537S and D538G cells were plated in triplicate and treated with an escalating dose of IR, and cell survival was assessed. Error bars denote SD of three independent experiments. **d**, **e** Immunofluorescence microscopy was used to quantify γ-H2AX foci formation in MCF-7 Y537S and D538G cells at 30 and 120 minutes after treatment with 4 Gy of IR in triplicate. Blue = DAPI. Green = γ-H2AX. 40 cells were counted in each independent experiment. **f**, **g** Alkaline comet assay of MCF-7 Y537S and D538G cells in triplicate following treatment with 4 Gy of IR and 30 minute recovery. 25 olive tail movements were measured per independent experiment. **e**, **g** the edges of the box denote first and third quartiles, the line denotes median, and the whiskers denote minimum and maximum derived from three independent experiments (*n* = 3). Statistical significance was evaluated using ANOVA with Dunnett’s test to adjust for multiple comparisons to wild-type sample. **p < 0.005, ***p < 0.0005.

Targeting pathological transcriptional^6^ and DNA repair reprogramming^7^ is emerging as a novel approach to overcome treatment resistance in many cancers. The bromodomain and extraterminal domain (BET) family of proteins such as BRD4 are epigenetic regulators that mediate *ESR1* mutation-induced “transcriptional addiction” in BC to confer ET resistance^8^. Using a high-throughput screen of nearly 1,200 drugs, we have previously identified OTX015, a BET inhibitor, as one of the top suppressors of *ESR1* mutant BC cell and xenograft growth^8^. Feng and co-workers have previously shown that BET inhibition overcomes tamoxifen resistance in BC by targeting cooperative interactions between BRD4 and WHSC1, a histone H3K36 methytransferase^9^. Thus, BET inhibition targets pathological transcriptional reprogramming in ER-positive BC to overcome ET resistance. We have previously shown that BRD4 also plays an essential role in DNA double-strand break (DSB) repair by engaging with several proteins involved in non-homologous end-joining repair pathway^10^. In prostate cancer, higher nuclear BRD4 protein expression in pre-treatment tumor samples is associated with higher rate of treatment failure in patients receiving RT as a part of primary treatment, suggesting that BRD4 plays an important role in RR^10^. Interestingly, we have observed that both MCF-7 and T-47D cells harboring *ESR1* Y537S and D538G mutations, as well as MCF-7 Tam-R cells, exhibit overexpression of BRD4 relative to the corresponding WT cells (Fig. 2a). Supported by these findings, we hypothesized that BRD4 mediates both ET resistance and RR in BC, and that pharmacologic BET inhibition overcomes RR (in addition to ET resistance as previously shown)^8,9^. To test this hypothesis, we conducted cell fractionation assays following IR treatment to evaluate the role of BRD4 in the repair of IR-induced DNA DSBs. Treatment of MCF-7 Y537S cells with IR resulted in enhanced acetylation of histone H4 and increased recruitment of BRD4 and other DNA repairs proteins such as 53BP1, KU80, and XRCC4 to the chromatin fraction (Fig. 2b). Pre-treatment with OTX015, a small molecule BET inhibitor^11^, significantly suppressed enrichment of these proteins in the chromatin fraction (Fig. 2b). OTX015 treatment also resulted in enhanced γ-H2AX foci formation and comet tail moment, suggesting that BET inhibition enhances IR-induced DNA damage (Fig. 2c–f and Supplementary Fig. 2a–d). Furthermore, OTX015 treatment reversed RR in both *ESR1* mutant cells and Tam-R cells and re-established susceptibility to IR (Fig. 3a, b and Supplementary Fig. 3a–d). We recapitulated these findings using a second BET inhibitor, JQ1, which reversed RR in both MCF-7 and T-47D *ESR1* mutant cells, and MCF-7 Tam-R cells (Supplementary Fig. 3e–j). Preliminary mechanistic investigations suggest that OTX015 overcomes RR by enhancing radiation-induced autophagy and apoptosis (Supplementary Fig. 3k, l). Thus, BRD4 plays a central role in mediating both transcriptional reprogramming that confers ET resistance^8,9^, and DNA repair reprogramming that confers RR in ER-positive BC cells.

**Fig. 2:**
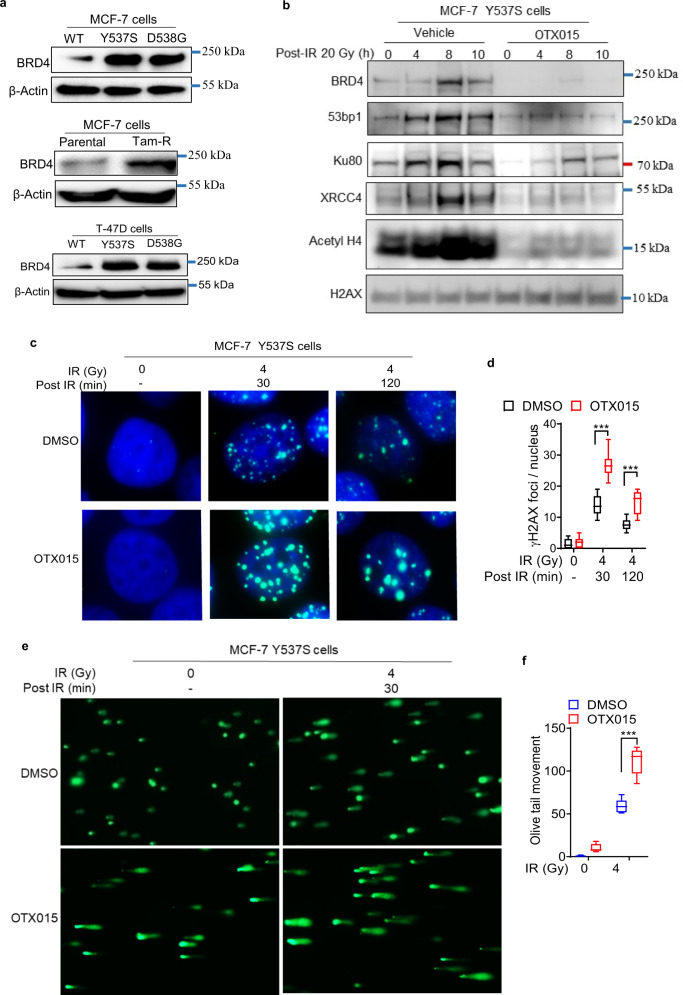
BRD4 mediates radiation response in ET-resistant breast cancer cells. **a** Western blot analysis was performed on whole-cell lysates derived from the indicated panel of ET-resistant cell lines using anti-BRD4 antibody. **b** MCF-7 Y537S cells were treated 20 Gy of radiation after pre-treatment with vehicle or OTX015 (1 μM). Cells were harvested at the indicated time points, subjected to chromatic fractionation, and analyzed by immunoblotting using the indicated antibodies. **c**, **d** Immunofluorescence microscopy was used to quantify γ-H2AX foci formation in MCF-7 Y537S cells at 30 and 120 minutes after exposure to 4 Gy of IR following treatment with vehicle or OTX015 (1 μM) in triplicate. Blue = DAPI. Green = γ-H2AX. 40 cells were counted per independent experiment. **e**, **f** Alkaline comet assay of MCF-7 Y537S cells after exposure to 4 Gy of IR and 30 minute recovery following treatment with vehicle or OTX015 (1 μM) in triplicate. 25 olive tail movements were measured per independent experiment. **d**, **f** The edges of the box, denote first and third quartiles, the line denotes median, and the whiskers denote minimum and maximum derived from three independent experiments (*n* = 3). Statistical significance was evaluated using an unpaired, two-tailed *t* test at the indicated time point. ***p < 0.0005.

**Fig. 3:**
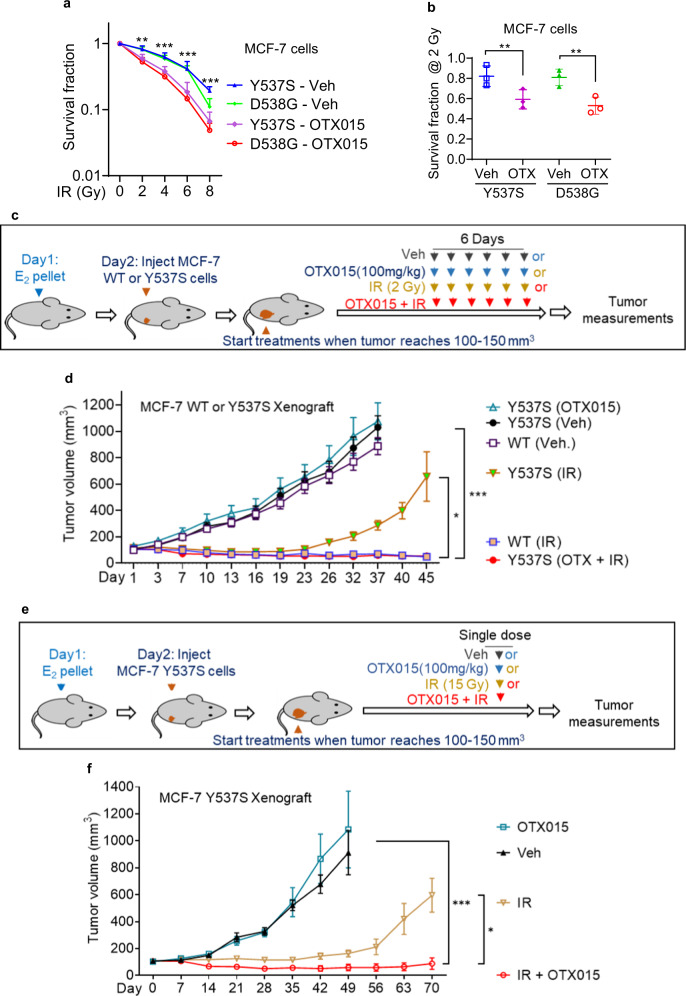
BET inhibition overcomes radiation resistance in ET-resistant breast cancer cells and xenografts. **a**, **b** ET-resistant MCF-7 Y537S and D538G cells were treated with an escalating dose of IR following pre-treatment with vehicle or OTX015 (1 μM) in triplicate, and cell survival was assessed. Survival of cells treated with IR + OTX015 was normalized to the survival of cells treated with OTX015 alone. Error bars denote SD of three independent experiments. **c**, **d** When tumors reached 100–150 mm^3^, mice bearing xenografts derived from MCF-7 WT cells or MCF-7 Y537S cells were treated with vehicle, OTX015 (100 mg/kg), IR (2 Gy x 6) or OTX015 + IR. Mice on OTX015 arm received OTX015 treatment on 6 consecutive days. Mice of OTX015 + IR received the drug on the days of IR for 6 consecutive days. Error bars denote SEM of *n* = 10 tumors per treatment arm. **e**, **f** When tumors reached 100–150 mm^3^, mice bearing xenografts derived from MCF-7 Y537S cells were treated with vehicle, OTX015 (100 mg/kg), IR (15 Gyx1) or OTX015 + IR. Mice on OTX015 arm received drug treatment once. Mice on OTX015 + IR received drug treatment once on the day of IR. Error bars denote SEM of *n* = 10 tumors per treatment arm. Statistical significance was evaluated using the unpaired, two-tailed *t* test for pairwise comparisons (**a**, **b**). ANOVA with Dunnett’s test was used to adjust for multiple comparisons of experimental arms to control arm (**d**, **f**). Non-parametric Wilcoxon rank sum test with Bonferroni correction was used for comparing treatment groups to each other (**d**, **f**). *p < 0.05; **p < 0.005, ***p < 0.0005.

Next, we sought to determine whether acquired ET resistance confers RR in vivo and whether pharmacological BET inhibition reverses such resistance. In mice bearing xenografts derived from MCF-7 cells harboring the wild type *ESR1*, tumor growth was well controlled with RT alone (2 Gy administered on 6 consecutive days for a total dose of 12 Gy) (Fig. 3c, d). However, xenografts harboring the Y537S mutation treated with the same dose of radiation exhibited profound RR and nearly reached the size of sham-irradiated mice with a lag of approximately one week. To isolate the radiosensitization effects of OTX015 from its systemic effects, we administered OTX015 only on the days of RT (Fig. 3c). As a control, we gave six daily doses of OTX015 without radiation (Fig. 3c), unlike previous studies which administered BET inhibitors daily over several weeks^8,9^. Under these conditions, OTX015 did not exert any tumor growth inhibition (Fig. 3d). Concurrent administration of OTX015 + RT, however, resulted in high efficacy and completely suppressed the growth of radioresistant Y537S tumors (Fig. 3d). Even with extended monitoring of mice for over 4 months, radioresistant Y537S xenografts treated with RT + OTX015 did not show evidence of recurrence, although radiosensitive wild-type xenografts treated with RT alone recurred after a lag of two months (Supplementary Fig. 4a). Western blot analysis of radioresistant Y537S tumors (which recurred within 4 weeks after radiation) showed high expression of BRD4 while radiosensitive WT tumors (which recurred ~3 months after radiation) showed minimal expression (Supplementary Fig. 4b). These findings suggest that *ESR1* mutations confer RR in vivo, and combination of RT with BET inhibition overcomes *ESR1* mutation-induced RR and affords durable local control.

Stereotactic ablative radiation therapy (SABR) has been shown to be effective in the treatment of cancers that are traditionally considered resistant to fractionated RT^12^. We have previously shown that SABR-based adjuvant RT can be safely delivered in early-stage BC patients^13,14^. Remarkably, xenografts derived from MCF-7 Y537S cells remained radioresistant to high dose of radiation (15 Gy) delivered in a single fraction (Fig. 3f). However, single administration of OTX015 prior to 15 Gy of IR completely reversed RR in these xenografts (Fig. 3f). Analysis of synergy by Loewe additivity model^15^ showed that combination of OTX015 showed high synergy with both fractionated radiation (synergy score: 171) and single fraction radiation (synergy score: 101) (Supplementary Fig. 4c, d). Supported by these findings, we conclude that ET-resistant breast tumors are resistant to both conventionally fractionated and hypofractionated radiotherapy, and that pharmacological BET inhibition overcomes ET resistance-induced RR, both in vitro and in vivo.

Pre-operative ET use in post-menopausal women with localized, ER-positive BC affords comparable rates of response and breast preservation, but lower toxicity relative to chemotherapy use^1^. The ongoing COVID-19 crisis has also increased the use of pre-operative ET in localized ER-positive BC patients due to pandemic-related delays in surgery^16^. Pre-operative ET exerts selective pressure on cancer cells and promotes the evolution and/or enrichment of pathogenic alternations such as *ESR1* mutations and other cellular adaptations^17^. In this study, we show ET resistance drives RR through reprogramming of DNA repair pathways. We also show that both OTX015 and JQ1 reverse RR in both *ESR1* mutant and Tam-R BC cells. Thus, BRD4 plays a dual role in mediating both ET resistance^8,9^, and RR in ER-positive BC cells by mediating reprogramming of transcriptional and DNA repair pathways, respectively. Our findings are also consistent with reports that Tam-R BC cells are resistant to DNA-damaging chemotherapeutic agents such as adriamycin and cisplatin^18^.

OTX015, the BET inhibitor employed in this study, has demonstrated a favorable safety profile as a single agent in a previously completed Phase Ib clinical trial^11^. Several additional BET inhibitors are currently in various phases of clinical development^19^. Since normal cells have more proficient DNA repair capabilities relative to cancer cells^20^, the exacerbation of radiation-induced DNA damage by BET inhibition is likely to be more lethal in cancer cells than in the surrounding normal tissues. This differential may establish a therapeutic window for the safe administration of this combination regimen in patients, although this remains to be established in future clinical trials.

Overall, our findings suggest that ET resistance in the pre-operative setting serves as a biomarker for diminished response to adjuvant RT, and that pharmacological BET inhibition reverses RR in such patients. Since we observed evidence of RR in both Tamoxifen-resistant and *ESR1* mutant BC models, our findings have broad implications in both pre-menopausal women (who are commonly treated with tamoxifen) and post-menopausal women (who are commonly treated with aromatase inhibitors, which create an estrogen-depleted environment and exert selective pressure for the evolution of *ESR1* mutations)^4^. The increasing use of “window of opportunity” studies to assess response to ET in the pre-operative setting^21^ will further facilitate personalization of radiation treatments in these patients based on their response to ET.

There is also increasing interest in employing SABR to deliver ablative radiation to metastatic sites in carefully selected patients with oligometastatic BC^22^. Since a significant sub-set of metastatic ER-positive BC patients harbor ET resistance (due to *ESR1* mutations or other cellular adaptations), our findings suggest that such patients may harbor radiation-resistant metastatic sites and may benefit from treatment with a combination of RT + BET inhibitor. Thus, we provide a therapeutic rationale for personalization of radiation treatments in BC patients based on their response to ET. We also provide a molecularly targeted approach for reversing RR in such patients. Thus, our study establishes a framework for advancing Precision Radiation Oncology in BC patients.

## Methods

### Cell culture

T-47D and MCF-7 cells were purchased from American Type Culture Collection (ATCC). T-47D cells were cultured in RPMI medium (Sigma-Aldrich) and MCF-7 cells were cultured in DMEM medium (Sigma-Aldrich) supplemented with 10% fetal bovine serum (Genesee Scientific) and 2% Penicillin–Streptomycin (Sigma-Aldrich) in a humidified incubator with 5% CO_2_. Generation of *ESR1* mutant (Y537S and D538G) MCF-7 and T-47D cells were previously described^8,23^. Tamoxifen-resistant MCF-7 cells were provided by Dr. Carlos Arteaga. Cells were assessed for their viability and counted with a Countess II FL Automated Cell Counter (Life Technologies, Gaithersburg, MD). The cells were regularly tested for Mycoplasma contamination with PlasmoTest (Invivogen).

### Antibodies

BRD4 (Cat #13440, Cell Signaling Technology; Cat #ab75898, Abcam), Acetylated Histone H4 (Cat #06-598, Millipore), H2AX (Cat #2595, Cell Signaling), γ-H2AX/ phosphorylated histone H2A.X (Ser139) (Cat #05-636, Millipore), 53BP1 (Cat #NB100-304, Novus), XRCC4 (Cat #SC-271087, Santa Cruz), LC3A/B (Cat #12741, Cell Signaling Technology), Alexa Fluor 488 conjugated anti-mouse antibody (Cat #A-21121, Thermo Fisher Scientific), β-Actin (Cat #3700, Cell Signaling Technology) and Ku80 (gift from Dr. Benjamin Chen, UT Southwestern Medical Center at Dallas).

### Chemicals/drugs

4-hydroxytamoxifen was purchased from Sigma. β-estradiol pellets (0.17 mg, 2-week release) for subcutaneous implantation were purchased from Innovation Research of America. OTX015 was purchased from Abmole.

### Irradiation and cell survival assays

Hormone depletion was carried out by washing the cells with phenol red-free IMEM (GIBCO Cat# A10488-01) without serum (five washings daily, 1 hour apart). Cells were cultured in phenol red-free IMEM supplemented with 5% charcoal-dextran-treated calf serum (Valley Biomedical Cat# BS3050) and 2% penicillin-streptomycin in a T150 flask. Cells were trypsinized and 1 × 10^4^ cells were seeded in 6-well tissue culture plates. 24 hours after treatment with vehicle or OTX015 (1 μM), cells were subjected to IR treatment (Mark 1 137Cs irradiator, J.L. Shepherd and Associates) at various doses as indicated in each figure. Cells were then allowed to grow in an incubator for 7 days. On day 7, cells were washed, fixed with 4% paraformaldehyde, stained with 0.5% crystal violet solution for 20 minutes, washed with water, and air dried. After photographic images of the plates were obtained, crystal violet was solubilized with 20% acetic acid and absorbance was quantified by spectrophotometry at 590 nm using a Spark 10 M multimode microplate reader (Tecan).

### Immunoblotting

Hormone-deprived cells were lysed with modified RIPA lysis buffer containing 50 mM Tris-HCl pH 7.5, 1% NP-40, 0.25% sodium deoxycholate, 150 mM NaCl, 1 mM EDTA, complete protease inhibitor cocktail and phosphatase inhibitor cocktail (Roche), resolved by SDS-PAGE, and transferred onto a PVDF membrane. The membranes were immunoblotted with antibodies against BRD4 (1:1000 dilution; Cat #13440, Cell Signaling Technology) and β-actin (1:4000 dilution; Cat #3700, Cell Signaling Technology). Immunoreactive proteins were detected using ECL super signal west femto substrate reagent (Thermo Scientific). All blots were derived from the same experiment and were processed in parallel.

### Chromatin fractionation assay

MCF-7 Y537S cells were irradiated 24 hours after treatment with vehicle or OTX015 (1 μM). Cells were harvested and suspended in fractionation buffer I (50 mM HEPES, pH 7.5, 150 mM NaCl, 1 mM EDTA, 5 mM sodium butyrate) supplemented with 0.2% Nonidet P-40 and protease inhibitors (complete, EDTA free, Roche). Following centrifugation at 1000 g for 5 minutes, the cytoplasmic supernatant was discarded, leaving behind the nuclear pellets. Nuclear pellets were washed with the same buffer and resuspended in fractionation buffer II (same as fractionation buffer I, except for 0.5% Nonidet P-40) and incubated for 40 minutes on ice. After determining the concentration of each sample by BCA protein assay kit (Cat #23225, Thermo Scientific), chromatin pellets were obtained by centrifugation for 15 minutes at maximum speed at 4 °C. The pellets were then subjected to acid extraction with 150 μl of 0.2 N HCl to enrich chromatin-bound proteins. Extracted proteins were boiled with LDS sample buffer (B0008, Thermo Fisher) and separated on 4–12% bolt-Tris plus gels (NW04120, Thermo Fisher) and blotted onto PVDF membranes (Millipore). Membranes were blocked for 1 hour in 5% non-fat dried milk in TBST and incubated with primary antibody (1:1000) overnight at 4 °C. After three washes with TBST, membranes were incubated for 1 hour with secondary antibodies (1:4000) in TBST. The immunoblots were processed by Supersignal West Pico chemiluminescence kit (34080, Thermo Scientific), and images were obtained by ChemiDoc Touch Imaging System (Bio-Rad) and processed by Image Lab Touch (Bio-Rad). The following antibodies were used: BRD4 (1:1000 dilution; Cat #ab75898, Abcam), Acetylated Histone H4 (1:1000 dilution; Cat #06-598, Millipore), H2AX (1:1000 dilution; Cat #2595, Cell Signaling), γ-H2AX (1:1000 dilution; Cat #05-636, Millipore), 53BP1 (1:1000 dilution; Cat #NB100-304, Novus), XRCC4 (1:1000 dilution; Cat #SC-271087, Santa Cruz), and Ku80 (1:1000 dilution; gift from Dr. Benjamin Chen at UT Southwestern). All blots were derived from the same experiment and were processed in parallel. Uncropped and unprocessed scans of blots were provided as Supplementary Figures 5–8.

### Immunofluorescence analysis

Indicated MCF-7 and T-47D cells (1 × 10^4^) were plated on coverslips and after overnight culture, cells were treated with 1 µM OTX015 or DMSO for 24 hours followed by IR (4 Gy). 30 minutes and 120 minutes after IR treatment, cells were fixed in 4% paraformaldehyde, permeabilized using PBS with 0.3% Triton-X, and blocked with 5% goat normal serum. The coverslips were incubated overnight with Ser139 phosphorylated histone H2AX (γH2AX) antibody (1:1000 dilution; Cat #05-636, Millipore), followed by Alexa Fluor 488 conjugated anti-mouse antibody (1:200 dilution; Cat# A-21121, Thermo Fisher Scientific). The nuclei were counterstained and coverslips were mounted using ProLong Gold Antifade Mounting media with DAPI (Thermo Fisher Scientific). Images were captured by fluorescence microscopy (Zeiss, #LSM 880) and foci in the nucleus (at least in 40 nuclei for each treatment group of the experiment) were counted manually and plotted as the number of foci per nucleus.

### Comet assay

Indicated MCF-7 and T-47D cells (1 × 10^4^) were plated on six-well plate. After overnight culture, cells were treated with 1 µM OTX015 or DMSO for 24 hours followed by IR (4 Gy). 30 minutes after IR treatment, cells were harvested, resuspended in ice-cold PBS (without Mg^2+^ and Ca^2+)^ and subjected to comet assay (OxiSelect Comet Assay kit, STA-351, Cell Biolabs, Inc) using single-cell agarose gel electrophoresis under alkaline conditions following the manufacturer’s protocol. Briefly, 2 × 10^3^ cells in ice-cold PBS were combined with 10 volumes of comet agarose. 75 µl of cell-agarose mixture was poured on the comet slide and allowed to solidify at 4 °C in the dark for 15 minutes. Comet slides were incubated in ice-cold lysis buffer for 30 minutes followed by 30 minutes in the alkaline solution (300 mM NaOH, 1 nM EDTA) at 4 °C in the dark. Alkaline electrophoresis was done for 20 minutes at 1 volt/cm. Slide were rinsed in pre-chilled de-ionized water and ice-cold 70% ethanol for 5 minutes. The slides were air-dried and 100 μl/well of diluted Vista Green DNA was added to the slide’s well. Comet images were taken by fluorescence microscopy (Zeiss) using FITC filter and analyzed by CASPlab software.

### Terminal deoxynucleotidyl transferase dUTP nick-end labeling (TUNEL) assay

5 × 10^4^ MCF-7 Y537S cells were cultured on a coverslip in a 6-well plate. The cells were treated with an 8 Gy dose of IR after pre-treatment with vehicle or OTX015 (1 μM) for one hour. 24 hours after IR treatment, cells were washed with ice-cold PBS and fixed with 4% paraformaldehyde. After permeabilization with 0.1% Triton X-100 in 0.1% sodium citrate, TUNEL assay was performed using the manufacturer’s instructions (In Situ Cell Death Detection Kit- Fluorescein, Cat # 11684795910, Roche). Coverslip with the processed cells was mounted on the slide using VECTASHIELD Antifade Mounting Medium with DAPI (Vector Lab). Images were taken using a fluorescence microscope (Zeiss, #LSM 880). The TUNEL-positive cells and total cells were counted to determine the percentage of TUNEL-positive cells. Statistical significance was determined by a two-tailed unpaired *t* test. Data represent mean ± SD from three independent experiments.

### Detection of autophagy by LC3A western blotting

1 × 10^6^ MCF-7 Y537S cells were cultured on a 35-mm dish. Cells were treated with 8 Gy of IR after pre-treatment with vehicle or OTX015 (1 μM) for 1 hour. 24 hours after IR treatment, cells were washed with ice-cold PBS and lysed with modified RIPA lysis buffer containing 50 mM Tris-HCl pH 7.5, 1% NP-40, 0.25% sodium deoxycholate, 150 mM NaCl, 1 mM EDTA, complete protease inhibitor cocktail and phosphatase inhibitor cocktail (Roche), resolved by SDS-PAGE, and transferred onto a PVDF membrane. The membrane was immunoblotted with antibodies against LC3-I/II (1:1000 dilution; Cat #12741, Cell Signaling Technology). LC3-I/II proteins were detected using ECL super signal west femto substrate reagent (Thermo Scientific). The experiment was repeated thrice and a representative image was shown.

### Xenograft studies

All animal experiments were performed in accordance with UT Southwestern institutional animal care and use committee (IACUC)-approved protocol. MCF-7 WT and Y537S mutant cells were suspended in 3:7 (volume) matrigel:DMEM (Fisher Scientific) to a final concentration of 5 × 10^7^ cells/ml. 100 μl cell-gel mixture was then injected sub-cutaneously into the flank of 6–8 week old ovariectomized, female athymic, nude mice (Charles River). One day before the injection of cells, a β-estradiol pellet (0.17 mg/pellet, 14 days release) was implanted subcutaneously at a site distant from injection site. Xenograft growth was monitored by measuring tumor size twice a week using Vernier calipers and tumor volume was calculated using following formula: *π*/6 × larger diameter × (smaller diameter)^2^. When tumors reached a size of 100–200 mm^3^, mice were randomized to the following treatment arms: vehicle, IR as indicated, OTX015 (100 mg/kg as oral gavage), or IR + OTX015 (100 mg/kg by oral gavage). IR treatments included 2 Gy × 6 on consecutive days or 15 Gy × 1, as indicated. For mice that received concurrent OTX015 with fractionated IR, OTX015 treatment (100 mg/kg) was only given on the days of radiation. For mice that received concurrent OTX015 with single fraction of IR (15 Gy), a single dose of drug (100 mg/kg) was administered on the day of IR. Xenograft growth was continuously monitored by measuring tumor size twice a week until mice were sacrificed.

### BRD4 western blot analysis in xenograft tumors

Fractionated RT-treated MCF-7 WT and Y537S mutant xenografts were collected 134 days and 45 days, respectively, following RT. Three representatives WT and Y537S xenograft tumor tissues of approximately matching sizes were lysed with modified RIPA lysis buffer containing 50 mM Tris-HCl pH 7.5, 1% NP-40, 0.25% sodium deoxycholate, 150 mM NaCl, 1 mM EDTA, complete protease inhibitor cocktail and phosphatase inhibitor cocktail (Roche), resolved by SDS-PAGE, and transferred onto a PVDF membrane. The membrane was immunoblotted with antibodies against BRD4 (1:1000 dilution; Cat #13440, Cell Signaling Technology), and β-Actin (1:4000 dilution; Cat #3700, Cell Signaling Technology). Immunoreactive proteins were detected using ECL super signal west femto substrate reagent (Thermo Scientific).

### Analysis for synergy

Analysis for synergy of treatment combinations was carried out with R package ‘Synergyfinder’ and the online application (https://synergyfinderplus.org). The degree of synergy scoring in combination studies was quantified based on the deviations between the observed and the expected combination responses. Thus, responses were classified as synergistic if the combined effect is higher than the expected responses (or synergy score values >0) or antagonistic if the combined effect is lower than the expected responses (or synergy score value <0). We implemented the Loewe additivity model to obtain the synergy score^15^.

### Reporting summary

Further information on research design is available in the Nature Research Reporting Summary linked to this article.

## Supplementary information

Supplementary material

REPORTING SUMMARY

## Data Availability

All data generated or analyzed during this study are included in this published article (and its supplementary information files).
