# Supplementary material for: Towards precision radiation oncology: endocrine therapy response as a biomarker for personalization of breast radiotherapy
Source: NPJ Precis Oncol. 2023 Jan 24;7:11. doi: 10.1038/s41698-023-00348-1 (PMC9873388; doi:10.1038/s41698-023-00348-1)
Supplement: Supplementary file 1 — Supplementary material [file 41698_2023_348_MOESM1_ESM.pdf]

SUPPLEMENTARY DATA

Towards Precision Radiation Oncology: Endocrine Therapy Resistance as a Biomarker for Radiation Resistance in ER-positive Breast Cancer

SM Nashir Udden, GuemHee Baek, Kamal Pandey, Chantal Vidal, Yulun Liu, Asal S. Rahimi, D. Nathan Kim, Chika R. Nwachukwu, Ram S. Mani, and Prasanna G. Alluri

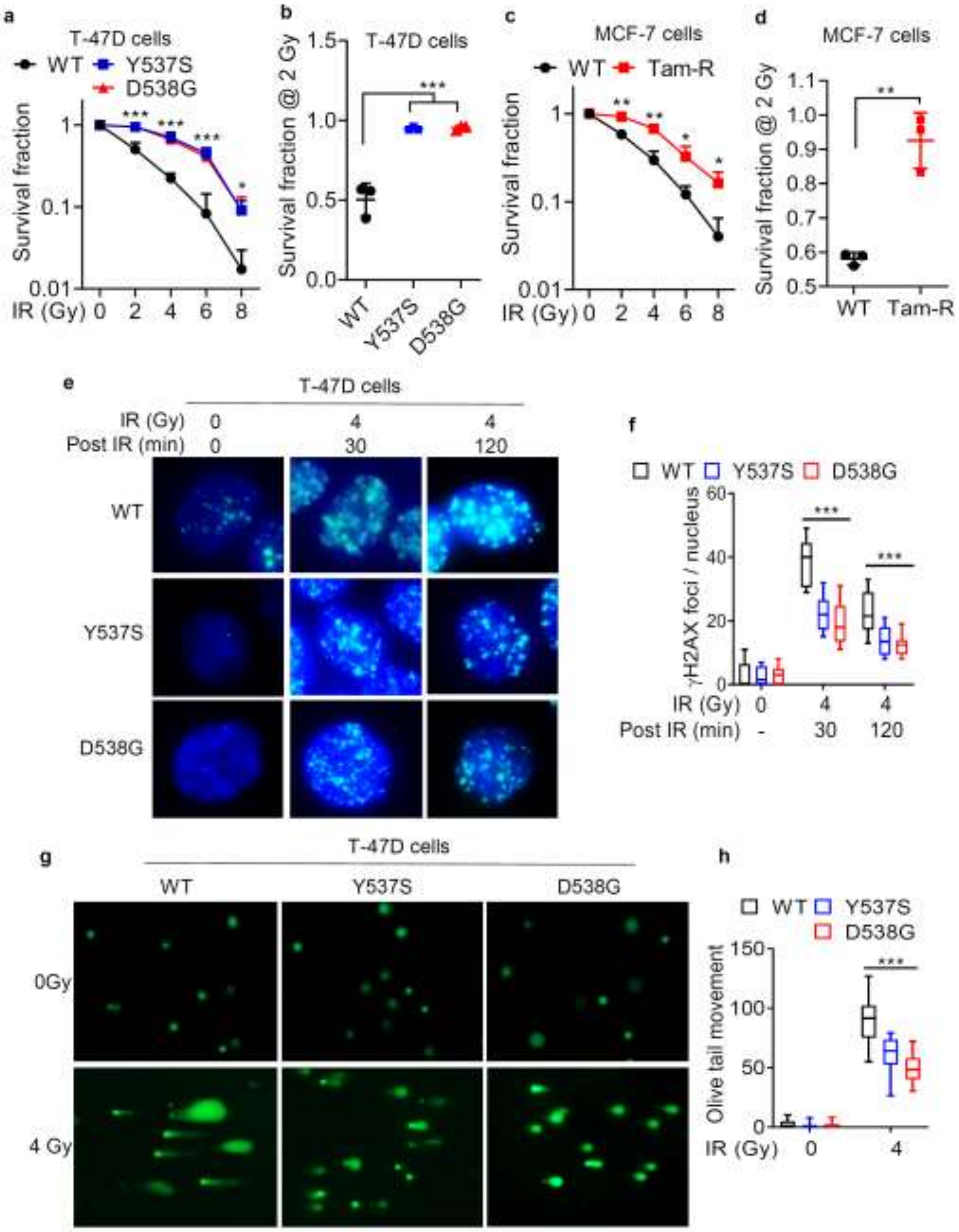

Supplementary Figure 1. ET-resistant T-47D and MCF-7 breast cancer cells exhibit radiation resistance. a-d: ET-resistant T-47D Y537S and D538G cells (a-b), and MCF-7 Tam-R cells (c-d) were treated with escalating dose of IR in triplicate and cell survival was assessed. Error bar denotes SD of three independent experiments. e-f: Immunofluorescence microscopy was used to quantify γ-H2AX foci formation in T-47D Y537S and D538G cells at 30 and 120 minutes after treatment with 4 Gy of IR in triplicate. Blue = DAPI, Green = γ-H2AX. 40 cells were counted per independent experiment. g-h: Alkaline comet assay of T-47D Y537S and D538G cells following treatment with 4 Gy of IR and 30 minute recovery in triplicate. 25 olive tail movement were measured per independent experiment. For f&h, the edges of the box denote first and third quartiles, the line denotes median, and the whiskers denote minimum and maximum derived from three independent experiments (n=3). Statistical significance was evaluated using an unpaired, two-tailed t test for pairwise comparisons (c&d); or ANOVA with Dunnett's test to adjust for multiple comparisons (a, b, e-h). * $p < 0.05$; ** $p < 0.005$, *** $p < 0.0005$.

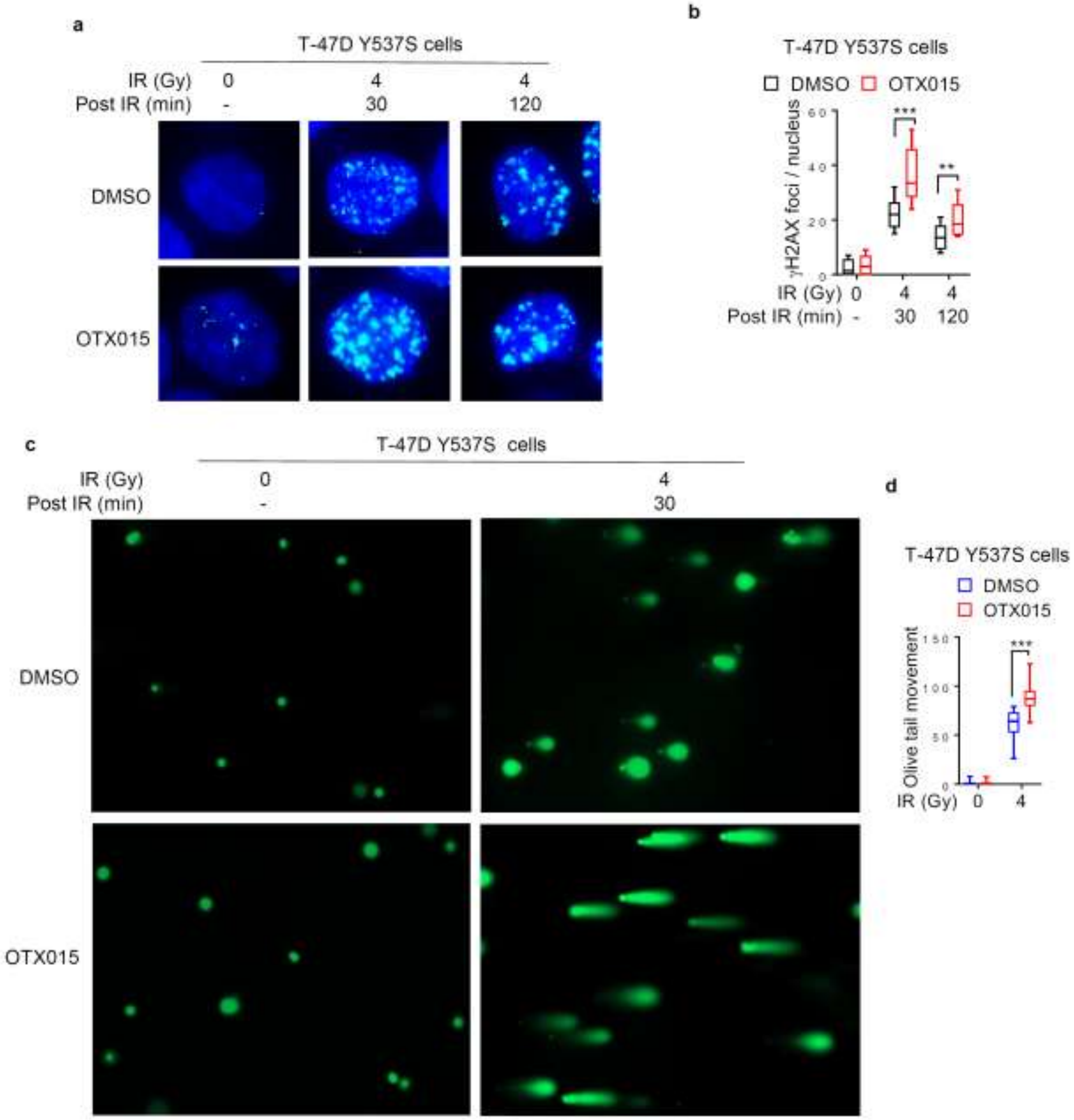

Supplementary Figure 2. Effect of OTX015 on unrepaired residual DNA damage after exposure to IR. a-b: Immunofluorescence microscopy was used to quantify γ-H2AX foci formation in T-47D Y537S cells at 30 and 120 minutes after exposure to 4 Gy of IR following treatment with vehicle or OTX015 (1μm) in triplicate. Blue = DAPI, Green = γ-H2AX. 40 cells were counted per independent experiment. c-d: Alkaline comet assay of T-47D Y537S cells after exposure to 4 Gy of IR and 30 minute recovery following treatment with vehicle or OTX015 (1μM) in triplicate. 25 olive tail movement were measured per independent experiment. For b&d, the edges of the box denote first and third quartiles, the line denotes median, and the whiskers denote minimum and maximum derived from three independent experiments (n=3). Statistical significance was evaluated using an unpaired, two-tailed t test at the indicated time point. ** $p < 0.005$; *** $p < 0.0005$.

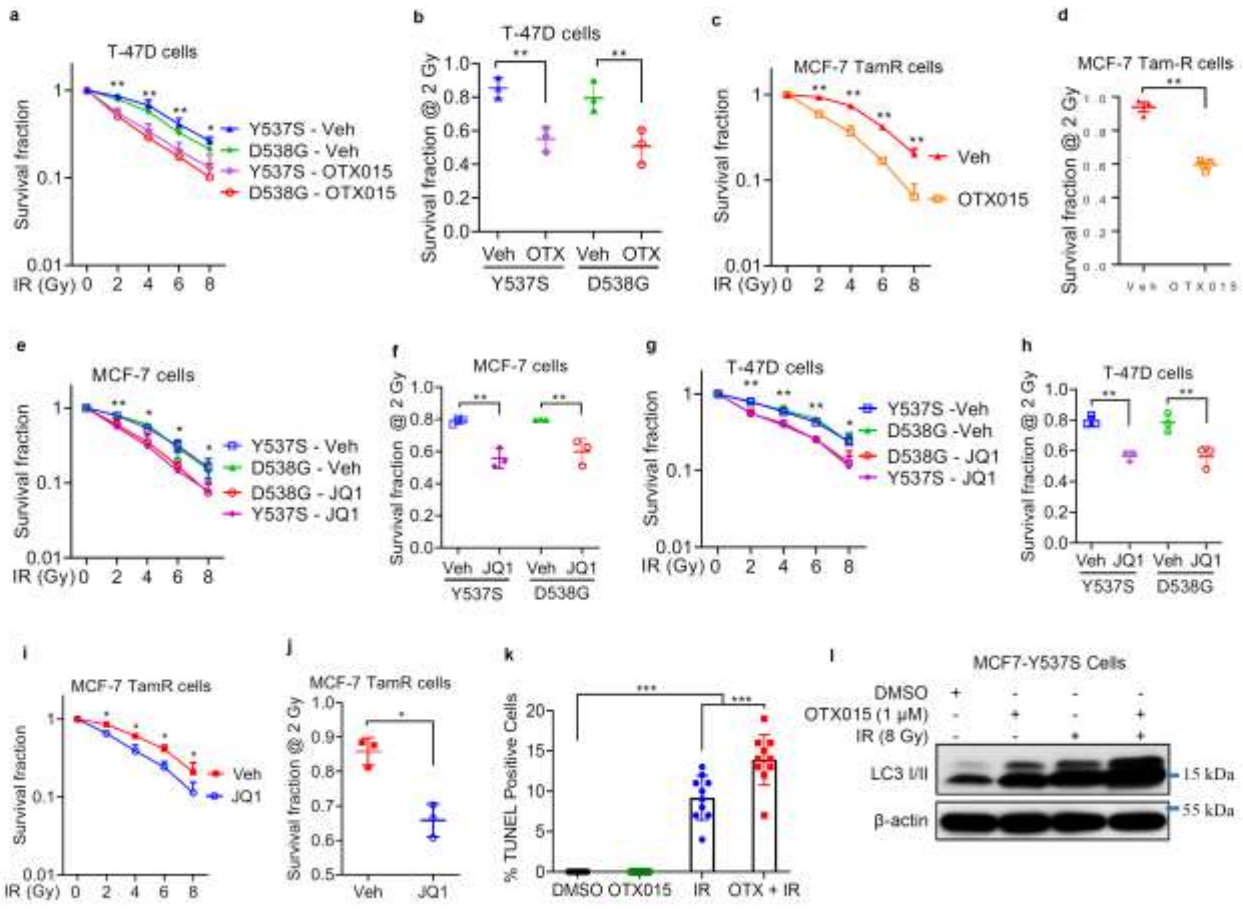

Supplementary Figure 3. BET inhibition overcomes radiation resistance in ET-resistant breast cancer cells. a-j: ET-resistant T-47D Y537S, D538G or MCF-7 Tam-R cells were treated with escalating dose of IR following pre-treatment with vehicle, OTX015 (1μM) or JQ1 (1μM) in triplicate and cell survival was assessed. Survival of cells treated with IR + OTX015 was normalized to survival of cells treated with OTX015 alone. Error bar denotes SD of three independent experiments. k: MCF-7 Y537S cells were treated with vehicle, OTX015 (1μM), IR (8 Gy)+vehicle, or IR (8 Gy)+OTX015 (1μM) in triplicate and percentage of Terminal deoxynucleotidyl transferase dUTP Nick End Labeling (TUNEL) positive cells were quantified using fluorescence microscopy 24 hours later. Error bar denotes SD of three independent experiments. l: MCF-7 Y537S cells were treated with vehicle, OTX015 (1μM), IR (8 Gy)+vehicle or IR (8 Gy)+OTX015 (1μM). Whole cell lysates were prepared 24 hours later and Western blot analysis was performed using anti-LC3 I/II antibody.

Statistical significance was evaluated using an unpaired, two-tailed t test for pairwise comparisons (a-j); or ANOVA with Dunnett's test to adjust for multiple comparisons (k). * $p < 0.05$; ** $p < 0.005$; *** $p < 0.0005$.

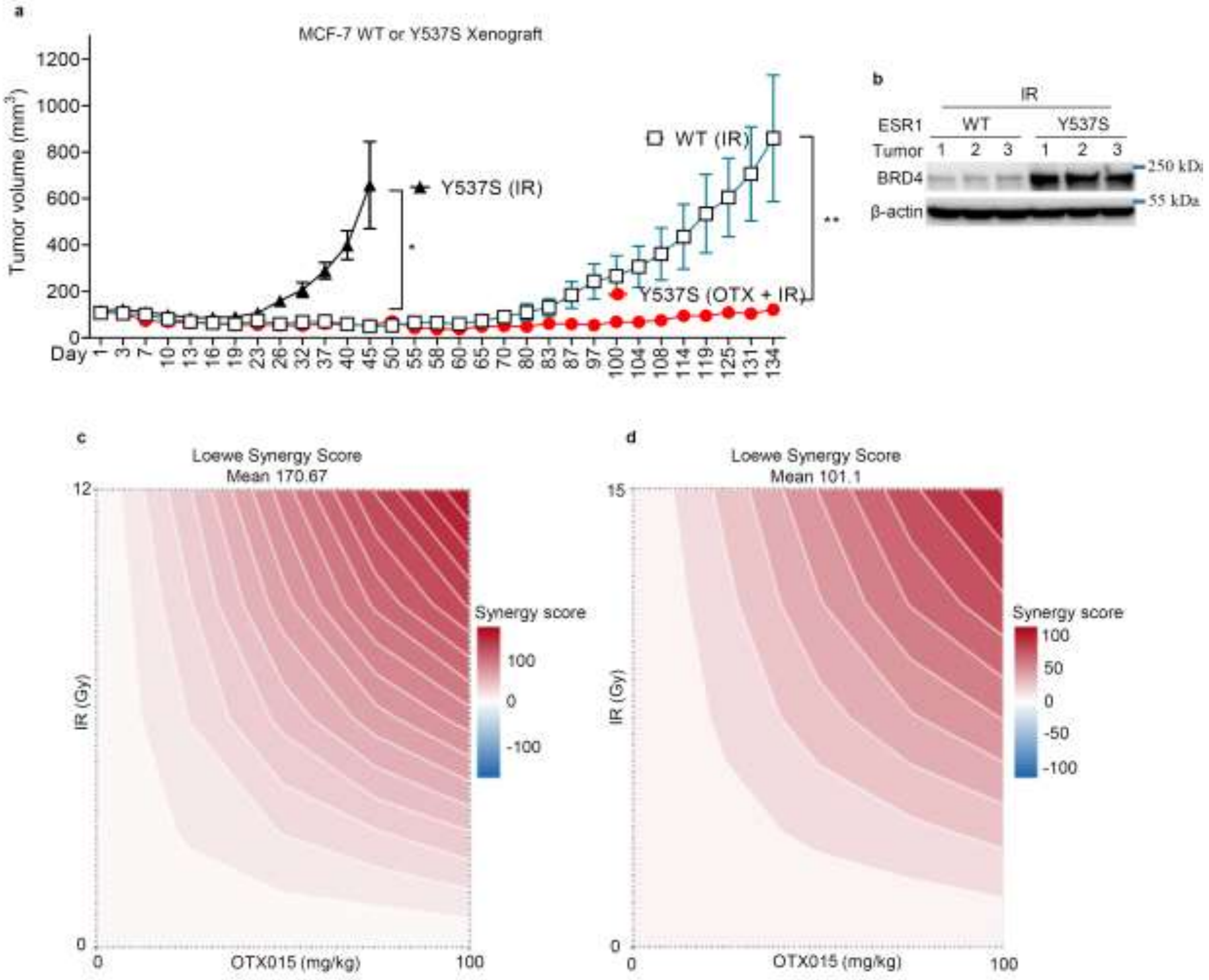

Supplementary Figure 4. OTX015 synergizes with RT to inhibit the growth of ET-resistant xenografts. a: MCF-7 WT tumors treated with IR, and MCF-7 Y537S tumors treated with IR or IR+OTX015 (as shown in Figure 3b) were followed for > 4 months. Radiosensitive WT tumors recurred 2 months after IR treatment while radioresistant Y537S tumors treated with IR+OTX015 showed durable control. Error bars denote SEM of n= 10 tumors per treatment arm. Statistical significance was evaluated using unpaired, two-tailed t test at the indicated time point. * =p < 0.05; ** = p < 0.005. b: Whole cell lysates were prepared from three representative radiosensitive WT tumors and radioresistant Y537S tumors of approximately matching size and Western blot analysis was performed using anti-BRD4 antibody. c-d: Loewe additivity model was employed to assess synergy between OTX015 and fractionated RT (c) or single fraction RT (d).

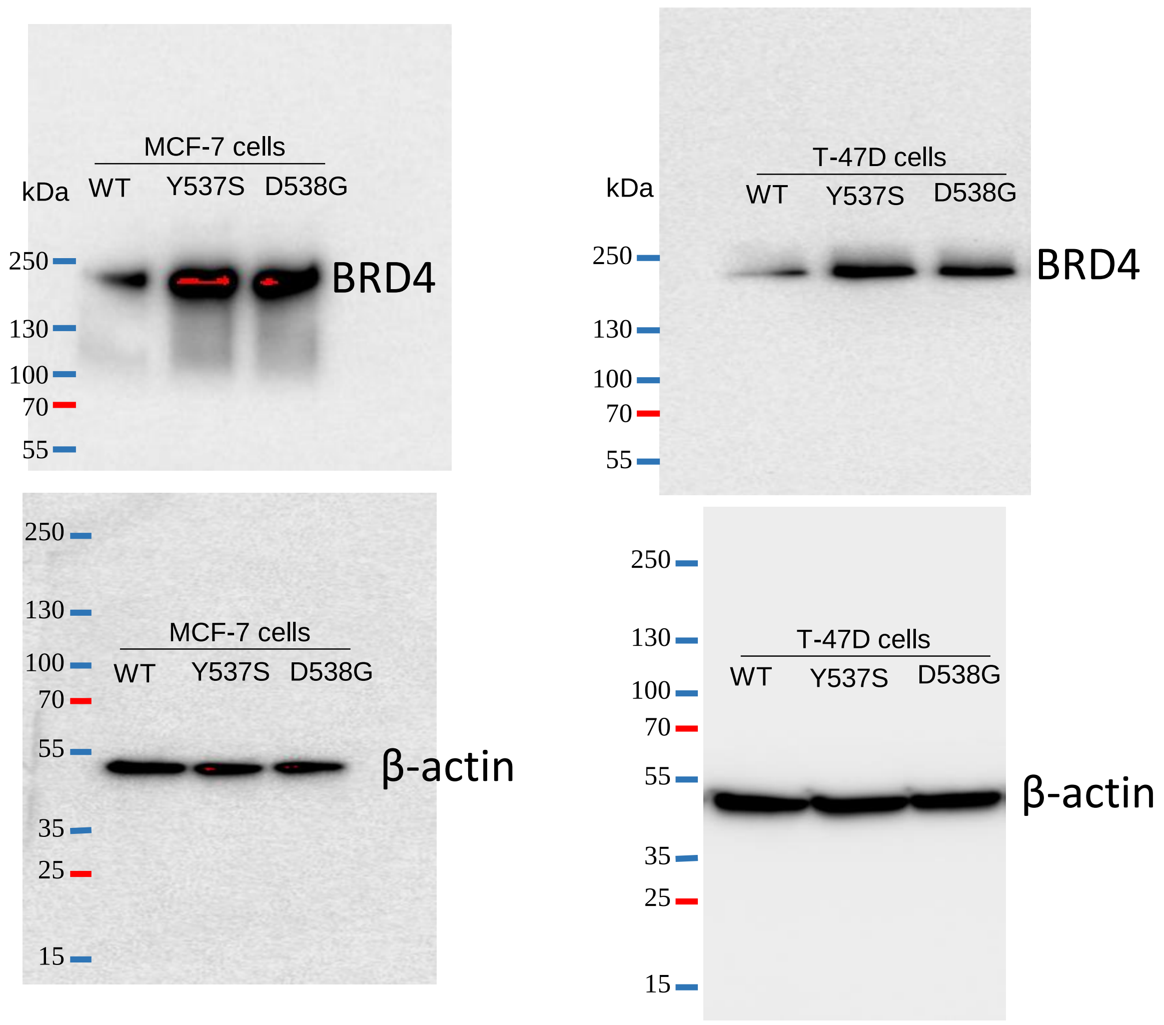

Supplementary Figure 5. Uncropped and unprocessed copy of blots corresponding to Fig. 2a.

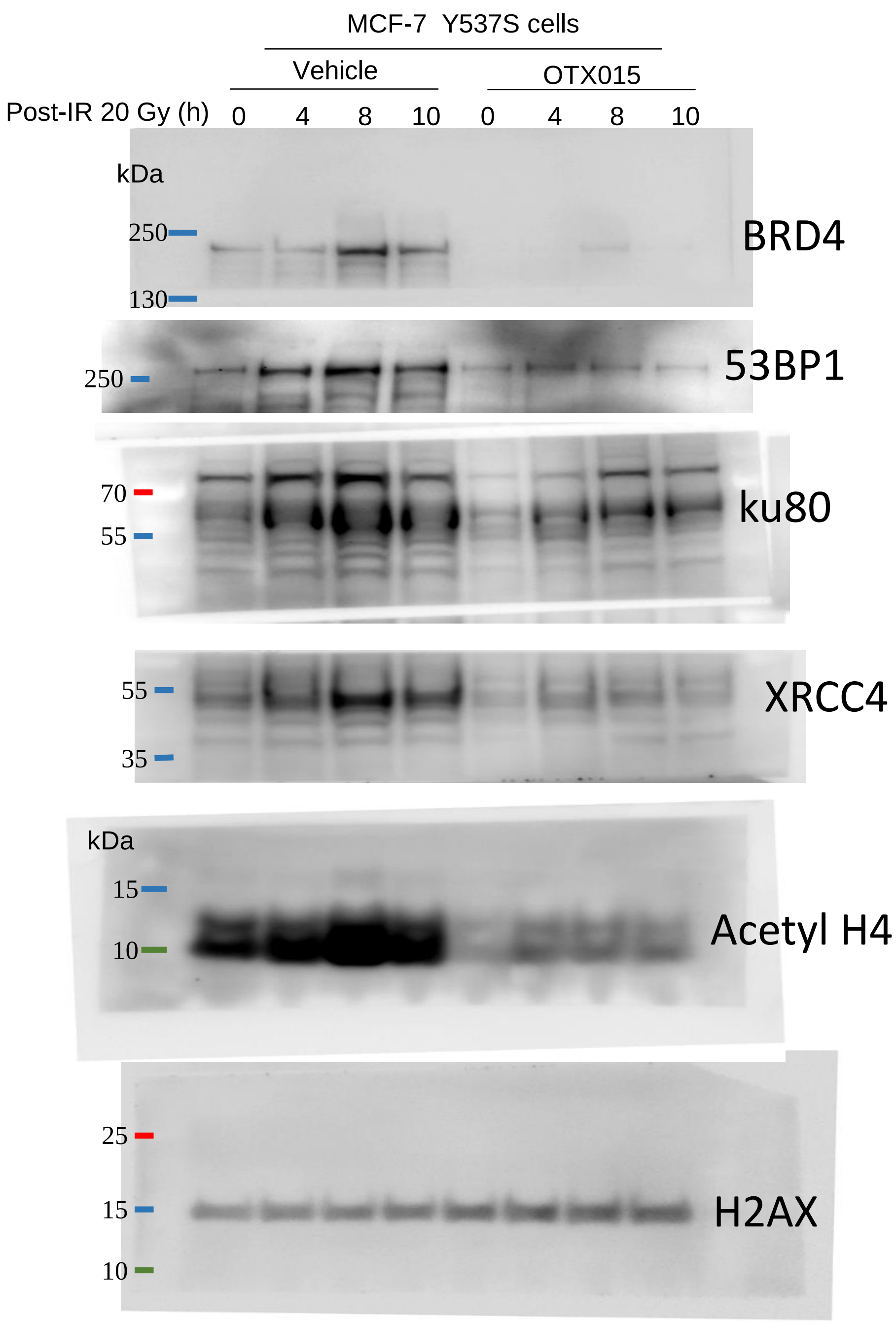

Supplementary Figure 6. Uncropped and unprocessed copy of blots corresponding to Fig. 2b.

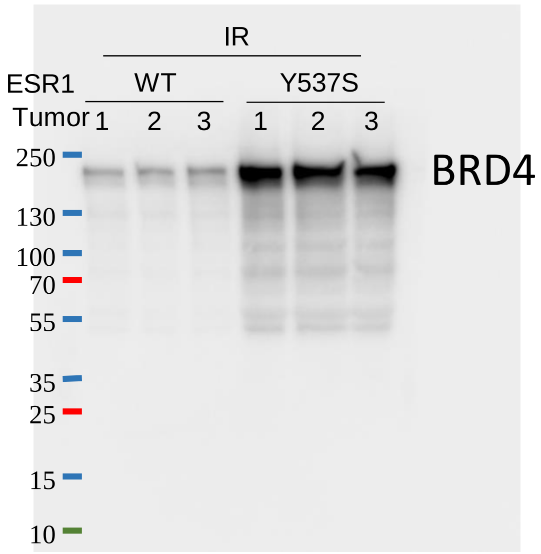

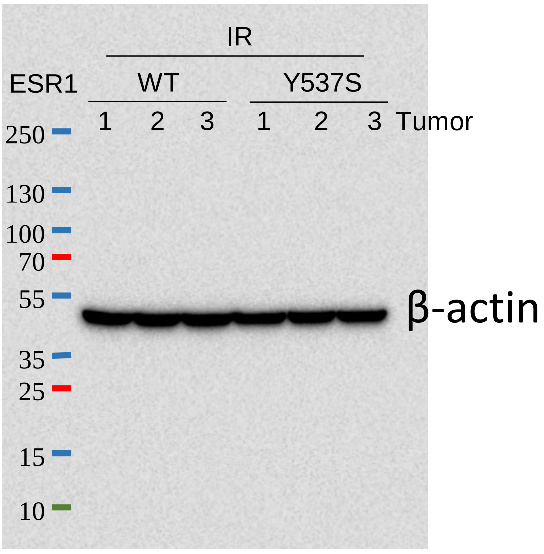

Supplementary Figure 7. Uncropped and unprocessed copy of blots corresponding to Supplementary Figure S3l.

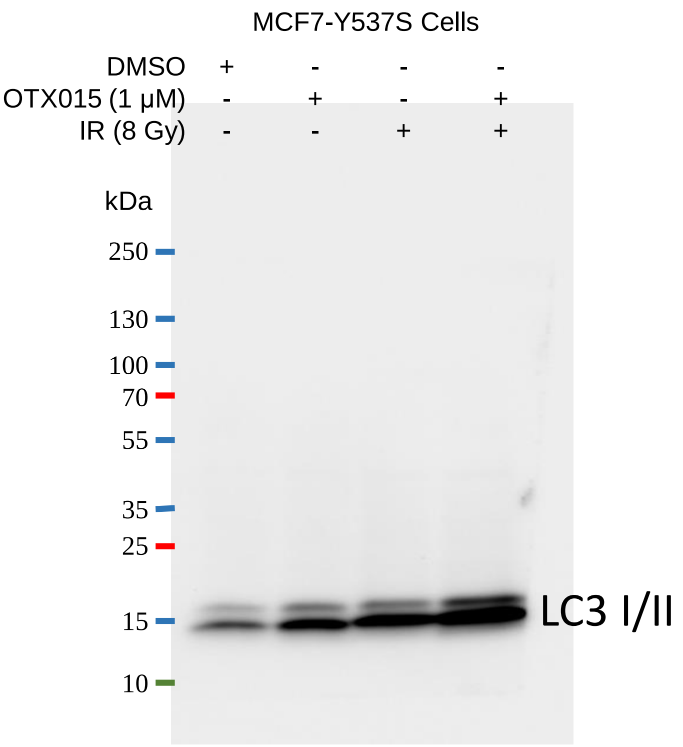

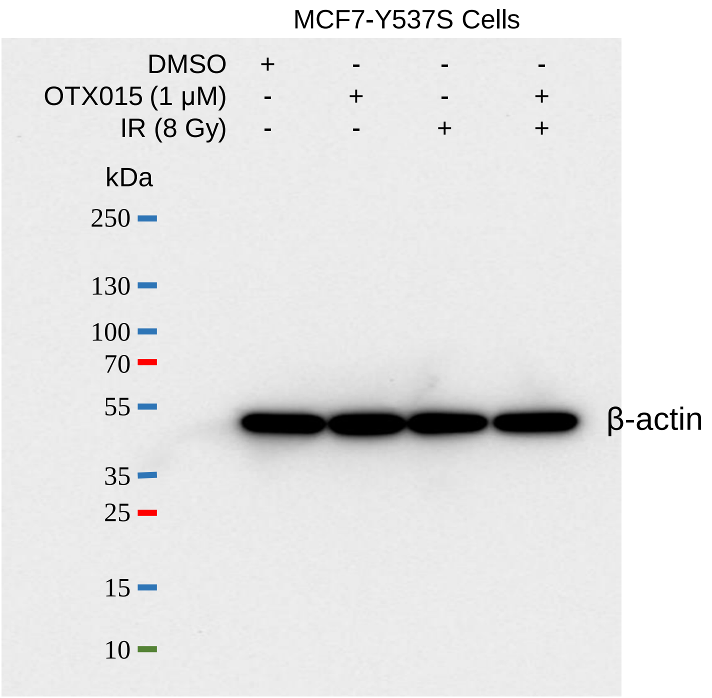

Supplementary Figure 8. Uncropped and unprocessed copy of blots corresponding to Supplementary Figure S4b.
