# Supplementary material for: Towards precision radiation oncology: endocrine therapy response as a biomarker for personalization of breast radiotherapy
Source: NPJ Precis Oncol. 2023 Jan 24;7:11. doi: 10.1038/s41698-023-00348-1 (PMC9873388; doi:10.1038/s41698-023-00348-1)
Supplement: Supplementary file 2 — REPORTING SUMMARY [file 41698_2023_348_MOESM2_ESM.pdf]

nature portfolio

Corresponding author(s): Dr Prasanna Alluri

Last updated by author(s): Jan 4, 2023

Reporting Summary

Nature Portfolio wishes to improve the reproducibility of the work that we publish. This form provides structure for consistency and transparency in reporting. For further information on Nature Portfolio policies, see our Editorial Policies and the Editorial Policy Checklist.

Statistics

For all statistical analyses, confirm that the following items are present in the figure legend, table legend, main text, or Methods section.

n/a	Confirmed	
☐	☒	The exact sample size (*n*) for each experimental group/condition, given as a discrete number and unit of measurement
☐	☒	A statement on whether measurements were taken from distinct samples or whether the same sample was measured repeatedly
☐	☒	The statistical test(s) used AND whether they are one- or two-sided<br>*Only common tests should be described solely by name; describe more complex techniques in the Methods section.*
☐	☒	A description of all covariates tested
☐	☒	A description of any assumptions or corrections, such as tests of normality and adjustment for multiple comparisons
☐	☒	A full description of the statistical parameters including central tendency (e.g. means) or other basic estimates (e.g. regression coefficient) AND variation (e.g. standard deviation) or associated estimates of uncertainty (e.g. confidence intervals)
☐	☒	For null hypothesis testing, the test statistic (e.g. *F*, *t*, *r*) with confidence intervals, effect sizes, degrees of freedom and *P* value noted<br>*Give P values as exact values whenever suitable.*
☒	☐	For Bayesian analysis, information on the choice of priors and Markov chain Monte Carlo settings
☒	☐	For hierarchical and complex designs, identification of the appropriate level for tests and full reporting of outcomes
☒	☐	Estimates of effect sizes (e.g. Cohen's *d*, Pearson's *r*), indicating how they were calculated

Our web collection on statistics for biologists contains articles on many of the points above.

Software and code

Policy information about availability of computer code

Data collection	Microsoft Excel 2016
Data analysis	GraphPad Prism 9

For manuscripts utilizing custom algorithms or software that are central to the research but not yet described in published literature, software must be made available to editors and reviewers. We strongly encourage code deposition in a community repository (e.g. GitHub). See the Nature Portfolio guidelines for submitting code & software for further information.

Data

Policy information about availability of data

All manuscripts must include a data availability statement. This statement should provide the following information, where applicable:

- Accession codes, unique identifiers, or web links for publicly available datasets
- A description of any restrictions on data availability
- For clinical datasets or third party data, please ensure that the statement adheres to our policy

All data generated or analysed during this study are included in this published article (and its supplementary information files).

Human research participants

Policy information about studies involving human research participants and Sex and Gender in Research.

Reporting on sex and gender	N/A
Population characteristics	N/A
Recruitment	N/A
Ethics oversight	N/A

Note that full information on the approval of the study protocol must also be provided in the manuscript.

Field-specific reporting

Please select the one below that is the best fit for your research. If you are not sure, read the appropriate sections before making your selection.

☒ Life sciences ☐ Behavioural & social sciences ☐ Ecological, evolutionary & environmental sciences

For a reference copy of the document with all sections, see nature.com/documents/nr-reporting-summary-flat.pdf

Life sciences study design

All studies must disclose on these points even when the disclosure is negative.

Sample size	10 mice per treatment group were used for xenograft studies
Data exclusions	No data were excluded
Replication	In vitro experiment was repeated 3 times as indicated in the manuscript
Randomization	When tumors reached 100-200 mm3, they were randomized to various treatment arms as indicated
Blinding	Investigators were blinded to treatment arm during data collection

Reporting for specific materials, systems and methods

We require information from authors about some types of materials, experimental systems and methods used in many studies. Here, indicate whether each material, system or method listed is relevant to your study. If you are not sure if a list item applies to your research, read the appropriate section before selecting a response.

Materials & experimental systems

n/a	Involved in the study	
☐	☒	Antibodies
☐	☒	Eukaryotic cell lines
☒	☐	Palaeontology and archaeology
☐	☒	Animals and other organisms
☒	☐	Clinical data
☒	☐	Dual use research of concern

Methods

n/a	Involved in the study	
☒	☐	ChIP-seq
☒	☐	Flow cytometry
☒	☐	MRI-based neuroimaging

Antibodies

Antibodies used	BRD4 (13440, Cell Signaling Technology; ab75898, Abcam), Acetylated Histone H4 (06-598, Millipore), H2AX (2595, Cell Signaling), [illegible]-H2AX/ phosphorylated histone H2A.X (Ser139) (05-636, Millipore), 53BP1 (NB100-304, Novus), XRCC4 (SC-271087, Santa Cruz), LC3A/B (12741, Cell Signaling Technology), Alexa Fluor 488 conjugated anti-mouse antibody (A-21121, Thermo Fisher Scientific), β-Actin (3700, Cell Signaling Technology) and Ku80 (gift from Dr. Benjamin Chen, UT Southwestern Medical Center at Dallas).
Validation	Commercially available antibodies were purchased from reputable sources and validated using a positive and negative control. Ku80 was received from Dr. Chen at UT Southwestern and specificity of the antibody was validated using siRNAs against Ku80.

Eukaryotic cell lines

Policy information about cell lines and Sex and Gender in Research

Cell line source(s)	T-47D and MCF-7 cells were purchased from American Type Culture Collection (ATTC). Generation of ESR1 mutant (Y537S and D538G) MCF-7 and T-47D cells were previously described (PMID: 28535794, PMID: 35881485). Tamoxifen-resistant MCF-7 cells were provided by Dr. Carlos Arteaga.
Authentication	The cells were authenticated by UT Southwestern genomics core facility using Short Tandem Repeat (STR) DNA profiling.
Mycoplasma contamination	The cells were regularly tested for any Mycoplasma presence with PlasmoTest (Invivogen).
Commonly misidentified lines (See ICLAC register)	NA

Animals and other research organisms

Policy information about studies involving animals; ARRIVE guidelines recommended for reporting animal research, and Sex and Gender in Research

Laboratory animals	6-8 week old, ovariectomized female athymic, nude mice were purchased from Charles River and maintained in a specific pathogen free (SPF) facility at UT Southwestern Medical Center.
Wild animals	N/A
Reporting on sex	Female mice were used as our studies involve breast cancer models
Field-collected samples	NA
Ethics oversight	IACUC

Note that full information on the approval of the study protocol must also be provided in the manuscript.
